# Wet Nursing and Human Milk Sharing: Reviving Sustainable Systems to Prioritise Breastfeeding

**DOI:** 10.1111/mcn.70144

**Published:** 2025-12-02

**Authors:** Anna Coutsoudis, Penny Reimers, John Cassey, Tanya Doherty

**Affiliations:** ^1^ Department of Paediatrics and Child Health University of Kwa Zulu Natal Durban South Africa; ^2^ MESCH—Medical and Educational Sustainable Community Help Newcastle Australia; ^3^ Health Systems Research Unit South African Medical Research Council Cape Town South Africa; ^4^ Department of Paediatrics and Child Health, Faculty of Health Sciences University of Cape Town Cape Town South Africa

**Keywords:** milk sharing, prioritising breastfeeding, sustainable support, wet nursing

## Abstract

The 2025 call of the World Alliance for Breastfeeding Action (WABA) is to prioritise breastfeeding through creating sustainable support systems. This is based on the strong foundations of breastfeeding and the unique properties of human milk to nourish, protect and provide optimal physical, emotional and cognitive growth for the infant. Empowering women to establish and maintain lactation is critical for the short‐ and long‐term health of mothers and infants; reducing infant and maternal morbidity; reducing healthcare costs; and building healthy societies. Studies have demonstrated this can be done effectively and sustainably using peer educators to provide support, knowledge and self‐efficacy to establish and maintain breastfeeding. However, rates of breastfeeding remain far below global targets. Safe and sustainable support options are needed for mothers who struggle with an insufficient milk supply, despite lactation support. These options include wet nursing and safe human milk sharing. When these are not possible/feasible, donor milk from human milk banks should be considered. Creating support systems requires investing in financial and human resources to protect, promote and support breastfeeding through revisiting these sustainable approaches.

## Summary

- Breast milk provides the optimal support for the healthy growth and development of infants and young children.
- Breastfeeding is environmentally sound and cost‐effective and contributes to a healthier human population.
- The 2025 World Alliance for Breastfeeding Action call for action is to prioritise breastfeeding through creating sustainable support systems.
- When mothers, despite lactation support, have challenges with breastfeeding, wet nursing or safe milk sharing are the optimal sustainable support strategies.
- Donor human milk should only be encouraged when other strategies are not feasible, especially in banks where milk is pasteurised, leading to some degradation of the milk.

## 1 Introduction

The World Alliance for Breastfeeding Action's (WABA) 2025 call to governments, international agencies, health systems, civil societies, communities and individuals is to *prioritise breastfeeding through creating sustainable support systems*.

Human milk is a unique tissue. Apart from its constantly replenishable and sustainable nutritional role, it contains substances which control infection, modulate immunity and colonise the infant microbiome (Kalbermatter et al. 2012). It is one of the most unique and sustainable natural products in the history of man. It is not a medical product in the strict sense of the word since it is not designed to compensate for failures in human health. Instead, it specifically nurtures and protects both infants' and mothers' health and development. By definition, it is not a commodity and should clearly be: NOT FOR SALE.

The WABA call is to prioritise breastfeeding, not merely the increased use of human milk. This important distinction values the multiple benefits of breastfeeding to the infant and mother. Examples include changes in breastmilk immune cells resulting from infant infection, reducing the severity and duration of the infant's illness, and enhanced maternal bonding and secure attachment, contributing to cognitive growth and the emotional well‐being of infants (Tomaszewska et al. 2023; Modak et al. 2023).

In addition to the decreased risk of contamination, suckling at the breast provides additional benefits such as optimal dental and craniofacial development, greater reduction in stress and additional benefits as mentioned earlier (Cudziło et al. 2018; Nagel et al. 2022).

Perhaps one of the most important advantages is the interaction of the infant's saliva with breastmilk, producing a combination of stimulatory and inhibitory metabolites that regulate early oral—and hence gut—microbiota. These interactions are therefore likely to play a part in boosting early innate immunity (Al‐Shehri et al. 2015). Use of water and electricity and often the exorbitant cost of donor human milk are also avoided.

Apart from short‐ and long‐term health benefits for infants/children, maternal benefits include reduction in postpartum depression and anxiety (due to increased secretion of the oxytocin hormone), as well as reduction in breast and ovarian cancer, hypertension, diabetes and cardiovascular disease. These could result in cost savings of billions of dollars as well as a reduction in maternal mortality rates (Doan et al. 2020).

United Nations Children's Fund (2018) summarised the impact of breastfeeding as follows:

In short, breastfeeding is among the most effective ways to protect maternal and child health and promote healthy growth and optimal development in early childhood. Empowering and enabling women to breastfeed should be at the heart of countries' efforts to keep every child alive and to build healthy, smart and productive societies.

Globally, only 48% of infants under 6 months are exclusively breastfed, with the global breastfeeding collective target set at 70% by 2030 (World Health Organization 2023).

## 2 What Interventions Work to Create Sustainable Support Systems?

The Lancet 2023 Breastfeeding Series address the policies and interventions needed to achieve optimal breastfeeding (Pérez‐Escamilla et al. 2023). An examination of reviews between 2016 and 2021 concluded that multilevel and multicomponent interventions across settings could quickly improve breastfeeding practices. The series framework outlines integrated interventions needed at three levels to improve breastfeeding. At the individual mother–infant level, counselling support and lactation management; within health systems, families and workplaces, legislation, policy, financing, monitoring and enforcement; and at a societal level, political mobilisation, social mobilisation and mass media.

There is strong evidence for the impact of peer or community health workers in improving rates of initiation and exclusive and any breastfeeding. A major challenge, however, is the small‐scale nature of many of these programmes and the lack of adequate investment by governments to ensure sustainability (Lewin et al. 2010).

A systematic review of randomised controlled trials looked at sustaining breastfeeding interventions and identified barriers and facilitators in low‐ and middle‐income countries (Engelhart et al. 2022). Important factors supporting sustainability included having motivated implementers, well‐funded supervision and versatile community health workers (CHWs). Barriers identified included minimal hours spent on the programme activities, insufficient and poorly paid CHWs, high costs, and importantly a lack of accountability.

The 2023 Lancet breastfeeding series reinforces that CHWs can amplify networks of breastfeeding education and support across healthcare, community and family settings and might be particularly helpful in complex situations like humanitarian emergencies (Pérez‐Escamilla et al. 2023).

Education and preparation for breastfeeding should begin antenatally. This can be done sustainably using peer educators, as has been done successfully in Mbale, Uganda. These educators followed up with every mother each day after delivery until they were discharged. If infants are born prematurely, mothers are supported to express and establish lactation (Magowan et al. 2020). Similarly, in an informal settlement in South Africa, mothers who were participants in a research study and were breastfeeding their infants were educated as peer supporters. They gained confidence and self‐efficacy and were confident enough even to question healthcare professionals at Child Health Clinics who were giving incorrect breastfeeding advice (Mulol and Coutsoudis 2018; Horwood et al. 2020).

Lactation supporters are vital from day 1 to support mothers to initiate and sustain breastfeeding even in the face of challenges. This should also include situations where a mother's milk supply dries up or she has decided to discontinue breastfeeding. Experienced lactation supporters can assist such women to re‐lactate (Amat Camacho et al. 2023; Bilgin and Karabayır 2024).

A review by the World Health Organization reported that re‐lactation is possible and practical for almost any woman if she is adequately motivated and supported. Success was higher when re‐lactation was initiated within a few weeks of weaning and when infants were younger than 6 months (World Health Organization 1998).

There is also experience of successful re‐lactation by mothers in an emergency setting who were supported to re‐lactate in a mass re‐lactation campaign (Dehkhoda et al. 2013). Re‐lactation has been shown to improve weight gain in preterm infants in combination with Kangaroo care (Salih 2018).

## 3 Sustainable Options for Mothers Who, Despite Lactation Support, Experience Insufficient Milk Supply

The Lancet breastfeeding series (2023) reported that nearly half of mothers globally self‐report insufficient milk (SRIM) as the primary reason for introducing commercial milk formula in the first few months of life and for prematurely stopping breastfeeding. SRIM can generally be prevented or addressed successfully with appropriate support (Pérez‐Escamilla et al. 2023). For women, who despite lactation support, experience insufficient milk supply, there are options to provide mothers with supplementation or alternatives to their own insufficient milk; however, none of these options are perfect and equivalent to the mother's feeding of her infant with raw human milk at the breast. Mothers need to be given time to understand and appreciate this and to receive continued support to produce their own milk, even while short‐term alternative options are put in place.

## 4 Options That Can Be Considered

### 4.1 DHMBs

This is cited by WHO and UNICEF as the next best alternative. It certainly protects the infant from sepsis and is more environmentally desirable than artificial cow's milk substitutes (World Health Organization 2024; Munblit et al. 2020).

There is enormous variation in how HMBs operate in different contexts, and these will not be discussed in detail as there is a large body of evidence and experience on human milk banks (HMBs). We note however the important salient points:

- Rendering donor human milk pathogen free through pasteurisation comes at the cost of total destruction of the normal microbiome
- Important immunological components, especially lactoferrin and IgA, are significantly impacted by some pasteurisation methods
- Lipase is completely destroyed—with implications for lipid absorption
- HMBs are typically associated with a neonatal intensive care unit in well‐resourced urban settings, with community supply being both limited and expensive.

### 4.2 Wet Nursing

Wet nursing, in the World Health Organisation hierarchy of best alternatives when breastfeeding is not possible, is listed after donor HMBs (DHMBs) (World Health Organization 2011). This seems paradoxical, especially in close community settings where breastfeeding is prevalent, and HIV is not prevalent. In these situations, wet nursing may be a more feasible alternative to donor human milk. Of ‘note’ in a country like Vietnam, where one of the authors (J.C.) has experience, there are many rural, low‐resourced communities where wet nursing would seem to be more appropriate than setting up expensive state‐of‐the‐art HMBs.

Wet nursing is promoted for emergency and humanitarian settings, and guidelines have been developed by UNICEF and NGOs, with success dependent on cultural acceptability and proper community engagement (United Nations Children's Fund UNICEF Infant Feeding in Emergencies Core Group 2025). Engagement with the community needs to be empathetic and respectful, using context‐specific approaches. The 2025 UNICEF technical and operational guidance on wet nursing offers practical steps on supporting wet nursing within health and nutrition emergency response programmes. Although intended for emergency response, the guidance is applicable in non‐emergency settings to strengthen access to human milk for vulnerable infants and normalise acceptance for wet nursing as an integral part of Infant and Young Child Feeding Policies (United Nations Children's Fund UNICEF Infant Feeding in Emergencies Core Group 2025).

Potential wet nurses would, in the first instance, be family members or neighbours/friends who are known to the mother. The wet nurses would have their health status screened in the same way a donor mother would, and, presumably, community health workers would know mothers well. Community‐based wet nurses will be exposed to similar pathogens and will have antibodies to these pathogens. Apart from the inherent benefits of breastfeeding referred to earlier, wet nursing, especially in emergency and disaster settings, means that the infant has no additional exposure to potential pathogens through expression or feeding using bottles, teats or cups. In addition, it enables a localised response with immediate accessibility without rigorous management requirements.

### 4.3 Safe Milk Sharing

The donation process is similar to DHMB except that the milk is not formally processed and pasteurised. However, donors are encouraged to be screened for similar lifestyle risks and where appropriate, depending on the epidemiological background of the community/region, appropriate laboratory tests should be performed (Sriraman et al. 2018).

The following points are of interest to mitigate the possible risk of milk sharing:

- Raw human milk, albeit from another mother, has all the advantages inherent in a mother's own raw milk as discussed earlier under the advantages of breastfeeding.
- Milk sharing between women in close proximity may alleviate the need for extra steps of freezing and thawing.
- Women in the same community will be exposed to similar pathogens and will build up antibodies to cope with this.

When resources are limited, donor mothers should ideally be taught to use hand expression since breast pumps are very difficult to clean adequately in that situation and feeding can be done from a cup. The hygiene conditions of the home should be known and assessed to be appropriate, and if the milk is to be expressed and stored before being provided to the infant, then refrigeration facilities need to be adequate. Furthermore, the donor mother should be known to the healthcare workers or the recipient mother (Keim et al. 2014). Informal sharing of milk through the internet or without donor screening and documentation of the collection and cold‐chain processes should be discouraged.

A mixed‐methods systematic review examined factors that facilitate and impede milk sharing and highlighted the need for further research, but emphasised that providing human milk to infants is an imperative public health action and all means of doing so require urgent action (Vickers et al. 2024).

### 4.4 Sustainable Support for Breastfeeding as a Humanitarian Priority

In emergencies and disasters, wet nursing, milk sharing and re‐lactation are likely to provide better outcomes for the infant than commercial milk formula and are concurrently environmentally friendly and cost‐effective (Smith 2019; Smith et al. 2023). In a recent model‐based analysis by Mowl, breastfeeding in humanitarian contexts showed substantial cost savings over commercial milk formula (Mowl et al. 2023). Unfortunately, the default response from NGOs and international aid organisations is usually to provide donations of commercial milk formula, with resultant early breastfeeding cessation and increased risk of morbidity and mortality (Hipgrave et al. 2012). Healthcare workers, especially in emergency settings, should, where appropriate, play a role in encouraging re‐lactation, milk sharing and wet nursing for those making decisions on infant feeding, thus assisting in the normalisation of wet nursing and milk sharing as appropriate strategies to protect breastfeeding.

Given the current state of war and conflict affecting many parts of the world, with resultant food insecurity and acute stress facing families, advocating for and investing in sustainable support systems for breastfeeding is imperative.

## 5 Conclusion

Safe and sustainable support options are needed for mothers who struggle with an insufficient milk supply, despite lactation support. These options include wet nursing and safe human milk sharing. When these are not possible/feasible, donor milk from HMBs should be considered.

Prioritising breastfeeding, including building robust and sustainable support systems for mothers, is an ideal solution contributing to a healthier human population. It saves lives, is environmentally sound and makes the best economic return on investment.

## Author Contributions

A.C. and P.R. wrote the first draft, and all authors contributed to the discussion and finalisation of the manuscript.

## Conflicts of Interest

The authors declare no conflicts of interest.

## Data Availability

Data sharing is not applicable to this article, as no new data were created or analyzed in this study.
